# Optimization of cold chain logistics distribution path considering traffic condition and replenishment along the way

**DOI:** 10.1371/journal.pone.0305982

**Published:** 2025-01-24

**Authors:** Wu Kai, Lu Zhijiang, Bai E.

**Affiliations:** Management School, Harbin University of Commerce, Harbin, Heilongjiang, China; Istinye University: Istinye Universitesi, TÜRKIYE

## Abstract

Road traffic congestion on the cold chain logistics not only increase the cost and time, but also creates certain negative impact on the national carbon emissions. To fully utilize the traffic resources, this study has classified urban road traffic congestion and defined the various vehicle delivery speeds with dynamic congestion levels. Simultaneously, it has developed the cold chain products replenishment strategy by considering delivery route, multi-depot condition and even vehicle types, aiming to minimize the total cost and carbon emissions, and maximizing the cold chain products freshness. To achieve this, this study build up a multi-objective vehicle routing optimization model and designed a hybrid algorithm combining large-scale neighborhood search and NAGA-II. Through computational analysis, this algorithm effectively overcomes the weak local search capability of NAGA-II and efficiently solves multi-objective problems. Moreover, under the simulated random traffic congestion conditions, this model able to demonstrate relatively stable planning results and address complex road traffic situations. Finally, this study able to analyze the impacts of various replenishment strategies, by considering multiple depots and sensitivity coefficients of cold chain products from delivery objectives. The analysis results also provides valuable insights for actual cold chain logistics distribution industry.

## 1 Introduction

As China leads with its strategic goals of achieving a carbon peak and carbon neutrality, low-carbon logistics has become a focal point for many scholars in cold chain research. Road traffic emissions are one of the main drivers in this field, as highlighted in reports by Stern [1] and Zhou [2]. Facing this challenge, there has been a surge in academic research aimed at reducing carbon emissions. Currently, in the field of low-carbon logistics, numerous scholars have incorporated carbon dioxide emissions as an optimization target in vehicle routing problems. Niu [3] established a green open vehicle routing problem with time windows based on the comprehensive modal emissions model (CMEM); Zhang [4] converted carbon emissions into costs and proposed an optimization model for low-carbon cold chain logistics. Jiang [5] developed a two-stage full vehicle distribution route optimization model that minimized transportation costs, refrigeration costs, and carbon emission costs. Li [6] proposed a multi-objective cold chain logistics distribution model considering the minimum carbon trading costs, which has been validated by solution. Xu [7] calculated carbon emissions using automotive fuel consumption, designing a mixed integer nonlinear programming model with objectives of fuel consumption and customer satisfaction.

These literature references are valuable for the optimization of vehicle carbon emissions. However, as Demir [8] points out, carbon emissions are directly related to vehicle fuel consumption. Typically, researchers replace fuel consumption rate with a constant, but in practice, the fuel consumption rate varies with vehicle speed, and road traffic conditions are usually the main reason affecting vehicle speed [9], hence giving rise to vehicle routing problems considering traffic congestion. Lu [10] used a three-tier speed function to simulate the speed restrictions on vehicles during peak hours and calculated the impact of traffic congestion on vehicle speed. Wang [11] established a mathematical model to optimize the order of node visits and paths between two nodes by considering the variation in vehicle speed across different road sections within the delivery area. Cai [12] developed a model that maintains good stability under different traffic congestion coefficients. Liu [13] divided the 24 hours of the day into equal time segments, each with different traffic congestion indices, termed as time-varying traffic congestion indices. Our research finds that incorporating time-varying traffic congestion indices into vehicle routing problems is more meaningful and practical.

In terms of replenishment strategies, Zhang [14] proposed three probabilistic models addressing the on-time delivery problem for vehicle routing problems with stochastic demand and time windows, with simulation results showing that replenishment strategies help achieve better solutions. Luca [15] considered random demand and replenishment in vehicle routing problems, finding that choosing replenishment strategies can improve overall transportation efficiency. Wang [16] introduced a novel multi-objective location routing problem, discovering that simultaneous pickup and delivery strategies could minimize distribution operation costs and maximize service levels. Qiu [17] considered the actual needs of simultaneous pickup and delivery, developing a multi-objective optimization model to address supply chain issues.

Furthermore, in optimizing the freshness of goods in cold chain logistics distribution, Wang [18] constructed a multi-objective distribution path optimization model that can better optimize the freshness of the goods. Helena [19] established a vehicle routing model related to time and temperature, reducing freshness loss due to temperature changes. Pérez [20] used theoretical probability distributions to simulate the lifespan of transported products for allocation within time windows. Additionally, regarding multiple depots and vehicle types, Laura [21] and Martins [22] explored the impact of multiple vehicle types on distribution efficiency, with results indicating that considering multiple vehicle types after conditions can reduce overall supply chain costs. Xu [23] developed a model based on multiple distribution centers, simulation results showing that it can further reduce the overall distribution time of vehicles.

In reality, pure cold chain logistics distribution is affected by traffic congestion, replenishment and delivery, customer satisfaction (freshness of goods), distribution distance, vehicle capacity, distribution centers and vehicle types, as well as delivery time. However, existing studies have not comprehensively considered all these factors in cold chain logistics. Thus, unlike [11, 13], we consider VRPs with replenishment strategies. Unlike [5, 6], we consider road traffic conditions in VRPs. Unlike [14, 15], we incorporate multiple depots and vehicle types into VRPs. Unlike [17, 21], we also consider freshness, establishing a multi-objective VRP. Table 1 introduces the features and contributions of our research.

**Table 1:** Abstracts of relevant literature.

Author	Traffic Congestion	replenishment	Carbon Emissions	Time Windows	Multi-Vehicle, Multi-Depot	Multi-Objective	Freshness
	With/Without	With/Without	With/Without	With/Without	With/Without	With/Without	With/Without
**Jiang et al. [5]**			√			√	
**Pérez et al. [20]**			√	√			√
**Wang et al. [11]**	√			√			
**Liu et al. [13]**	√		√	√			
**Li D et al. [6]**			√			√	√
**Xu B et al. [23]**			√	√	√	√	√
**Cai et al. [12]**	√	√	√	√		√	
**Luca et al. [15]**		√		√			
**Qiu et al. [17]**		√	√	√			
**Laura et al. [21]**				√	√		
**Zhang et al. [14]**		√		√		√	
**Martins et al. [22]**				√	√	√	
**This paper**	√	√	√	√	√	√	√

Motivated by research gaps, we aim to reduce the total cost, carbon emissions, and enhance the freshness of goods in cold chain logistics distribution during traffic congestion, by strategically planning multiple depots and vehicle types in cold chain logistics routes considering along-route replenishment and time windows. Our contributions are as follows:

First, we classify road traffic conditions and define the vehicle speeds under different congestion indices. Simultaneously, considering strategies for replenishment along the route, we constructed an optimization model for cold chain logistics distribution paths that include multiple depots and vehicle types, consisting of fixed costs, energy consumption costs, carbon emission costs, and refrigeration costs.

Second, we designed a multi-objective genetic algorithm combining Large Neighborhood Search (LNS) and NAGA-II (LNSNSGA-II), using a multi-chromosome coding method to build the initial sub-chromosomes. The classical genetic algorithm’s selection, crossover, mutation, and elitism preservation strategies are employed. After each iteration, local searches are conducted using the Large Neighborhood Search algorithm, and the Pareto front is updated. The effectiveness of LNSNSGA-II was demonstrated through comparative results from experimental simulations.

Finally, we conducted simulation experiments using the customer coordinate dataset R201 from Solomon’s VRP database. By simulating random traffic conditions, our model and algorithm demonstrated good stability. Additionally, by comparing strategies that do not consider replenishment and two different replenishment strategies, our cross-depot along-route replenishment strategy proved to be the best. Moreover, by comparing the number of distribution centers (depots), we found that multiple depots combined with along-route replenishment strategies can effectively reduce the costs and carbon emissions of cold chain delivery, and improve the overall freshness of goods. Lastly, we conducted a sensitivity analysis on the parameters of the goods freshness model in our study, finding that increasing the preservation effort level factor and reducing the time sensitivity factor can delay the impact of time on goods freshness. This provides a reference for enterprises in actual cold chain logistics distribution processes.

The remainder of this paper is structured as follows. Section 2 introduces the problem definition, including conditional assumptions, symbol definitions, and problem descriptions. Section 3 constructs a multi-objective cold chain logistics distribution model. Section 4 proposes the solution algorithms. Section 5 analyzes and discusses the simulation results. Section 6 concludes the paper. Section 7 provides an outlook on future research directions.

## 2 Problem definition

### 2.1 Conditional assumptions

(1) It is assumed that each depot (distribution center) has sufficient goods.

(2) It is assumed that there is only one type of fresh product, and the initial freshness of the fresh product when departing from the depot is the same.

(3) It is assumed that each depot has vehicles of multiple types, and the number of vehicles is limited.

(4) It is assumed that the vehicles at each depot can only serve customers within the service radius of that depot.

(5) It is assumed that the vehicles have limited driving distances due to energy constraints, but they will replenish energy when replenishing goods at depots along the route.

(6) The loading method of the goods is not considered, only whether the weight of the goods loaded on the vehicle exceeds the maximum load capacity of the vehicle.

### 2.2 Symbol definition

*p* = {1,2,3,…,*n*} represents the customer points, *V* = {*n*+1, *n*+2,…,*v*} represents the vehicles, *M* = {−1,−2,…,*m*} represents the depots, *G* = {*g*_1_, *g*_2_, *g*_3_} represents the vehicle types, where *g*_1_ and *g*_2_ represent fuel vehicles, and *g*_3_ represents electric vehicles. *v*_*g*_ represents the traveling speed of vehicles of type g, *m*_*g*_ represents the maximum load capacity of vehicles of type g, *L*_*g*_ represents the maximum travel distance of vehicles of type g, *c*_*g*_ represents the fixed dispatch cost of vehicles of type g, *fr*_*g*_ represents the unit time refrigeration cost during travel for vehicle type g, *fl*_*g*_ represents the unit time refrigeration cost for replenishing goods for vehicle type g, *need*_*n*_ represents the demand at customer point n, *t*_*n*_ represents the time the vehicle arrives at customer point n, *tti*_*n*_ represents the latest delivery time for customer point n, *Ti*_*n*_ represents the service time for customer point n, bt represents the time for vehicles to replenish goods at depots along the route, *R*_*n*_ represents the service radius of depot n, *k*_1_ represents the unit fuel consumption cost for fuel vehicles, *k*_2_ represents the unit travel distance cost for electric vehicles, *k*_3_ represents the unit carbon emission cost, *z*_*h*_ represents the traffic congestion index within time period h, ${load}_{v}^{h}$ represents the real-time load of vehicle v within time period h, *π*_0_ represents the initial freshness of the fresh product when leaving the depot, *l*_*ij*_ represents the distance between customer points i and j, τ represents the coefficient of preservation effort level (0<τ<1), α represents the time sensitivity factor of freshness (0<α<1), β represents the preservation effort sensitivity factor of freshness (0<β<1). The decision variables $x_{ij}^{v}=1$ indicates that vehicle v travels from node i to j (i≠j), otherwise $x_{ij}^{v}=0$; the decision variable $y_{i}^{v}=1$ indicates that node i is served by vehicle v, otherwise $y_{i}^{v}=0$; the decision variable $Z_{v}^{g}=1$ indicates that vehicle v is of type g, otherwise $Z_{v}^{g}=0$.

### 2.3 Problem description

There are m depots (distribution centers) and n customers in the delivery area. Each customer has a latest delivery time. There are v vehicles in total across all depots, and these vehicles come in three types: *g*_1_, *g*_2_ and *g*_3_. The first two types are fuel vehicles (which generate carbon emissions and are affected by traffic conditions), while the last type is electric vehicles (which do not generate carbon emissions and are not affected by traffic conditions). Due to the changing road traffic conditions, the delivery speed of vehicles will also change. At the same time, the freshness of the fresh products will decrease with the passage of time. The requirement is to minimize the total delivery cost, minimize the carbon emissions generated during the delivery process, and maximize the average freshness of fresh products delivered to all customer points. This must be achieved by considering the delivery mode of replenishing goods at depots along the route and finding a delivery route that meets these objectives.

## 3 Model construction

### 3.1 Traffic condition classification and definition of vehicle speed

The traffic congestion index *z*_*h*_ is classified into levels 0–10, where a higher index indicates a higher level of congestion. Considering that traffic congestion varies with time, we set the start service time *T*_*r*_ and end service time *T*_*l*_ for the depots. We divide the time period from *T*_*r*_ to *T*_*l*_ into u intervals, as calculated in Eq (1), Where tu represents the length of time intervals evenly divided from *T*_*r*_ to *T*_*l*_.


$$u=\left|\frac{T_{l}-T_{r}}{tu}\right|,$$ (1)

Therefore, based on reference [24], the travel speed of vehicle v of type g from customer point i to j under different traffic congestion index *z*_*h*_ is defined as $v_{ijgh}=v_{g}(1-\vartheta (z_{h}))$, where the calculation is as follows:

$$\vartheta (z_{h})=\{\begin{array}{l} 0,\;0\leq z_{h}<2\\ 0.0663z_{h}-0.1326,2\leq z_{h}<2\\ 0.0441z_{h}-0.0438,4\leq z_{h}<6\\ 0.0678z_{h}-0.1860,6\leq z_{h}<8\\ 0.0369z_{h}+0.0612,8\leq z_{h}<10 \end{array},h\in u,$$ (2)

### 3.2 Construction of the cold chain distribution model

#### 3.2.1 Objective analysis

(1) Fixed Cost *f*_1_: The fixed cost mainly consists of the fixed expenses incurred each time a vehicle starts (including irreversible wear and tear, acquisition costs, and related maintenance costs).


$$f_{1}=\sum_{g\in G}\sum_{v\in V}\sum_{m\in M}\sum_{i\in P}c_{g}x_{im}^{v}{y_{i}^{v}Z}_{v}^{g},$$ (3)

(2) Energy Cost *f*_2_: In this study, vehicles are classified into fuel vehicles and electric vehicles for the delivery task. The energy cost consists of fuel consumption cost $f_{2}^{\prime }$ for fuel vehicles and power consumption cost $f_{2}^{\prime \prime }$ for electric vehicles. Only fuel vehicles generate carbon emissions, while electric vehicles consume electricity without generating carbon emissions.

First, calculate the carbon emissions. The carbon emission rate *w*_*ijgh*_ for a vehicle v traveling at the speed *v*_*ijg*_ on a road (i, j) with zero gradient during time period h is defined as:

$$w_{ijgh}=\varphi_{0}+\varphi_{1}v_{ijgh}+\varphi_{2}v_{ijgh}^{2}+\varphi_{3}v_{ijgh}^{3}+\frac{\varphi_{4}}{v_{ijgh}}+\frac{{\varphi_{5}}^{2}}{v_{ijgh}}+\frac{{\varphi_{6}}^{3}}{v_{ijgh}},$$ (4)

where *φ*_0_, *φ*_1_, *φ*_2_, *φ*_3_, *φ*_4_, *φ*_5_, *φ*_6_ are parameters for the carbon emission rate of the vehicle, based on the vehicle type. The load correction factor ∅_*ijgh*_ for the vehicle v traveling on the road (i, j) during time period h is defined as:

$$\emptyset_{ijgh}=\beta_{0}+\beta_{1}\theta +\beta_{2}\theta^{2}+\beta_{3}\theta^{3}+\beta_{4}v_{ijgh}+\beta_{5}v_{ijgh}^{2}+\beta_{6}v_{ijgh}^{3}+\frac{\beta_{7}}{v_{ijgh}},$$ (5)

where *β*_0_, *β*_1_, *β*_2_, *β*_3_, *β*_4_, *β*_5_, *β*_6_, *β*_7_ are correction parameters for the load of fuel vehicles, and θ is the ratio of real-time load to maximum load of vehicle v of type g during time period h:

$$\theta =\frac{{load}_{v}^{h}}{m_{g}},$$ (6)

Therefore, the carbon emission rate for a vehicle v traveling on the road (i, j) during time period h is given by:

$$\varepsilon_{ijgh}=\frac{w_{ijgh}\emptyset_{ijgh}}{1000},$$ (7)

The carbon emissions for fuel vehicles are calculated as follows:

$$m_{{co}_{2}}=\sum_{h\in u}\sum_{g\in {\{g}_{1},g_{2}\}}\sum_{v\in V}\sum_{j\in \{p\cup M\}}\sum_{i\in \{p\cup M\}}x_{ij}^{v}{y_{i}^{v}Z}_{v}^{g}\varepsilon_{ijgh}v_{ijgh}(t_{j}-t_{i}),$$ (8)

Based on reference [25], 1 liter of gasoline produces 2.3 kg of carbon emissions. The fuel consumption rate for vehicle v during time period h is *f*_*ijgh*_ = *ε*_*ijgh*_/2.3, c3 represents the cost of 1 liter of fuel. The fuel consumption cost $f_{2}^{\prime }$ for fuel vehicles is calculated as:

$$f_{2}^{\prime }=k_{1}\sum_{h\in u}\sum_{g\in g_{1},g_{2}}\sum_{v\in V}\sum_{j\in \{p\cup M\}}\sum_{i\in \{p\cup M\}}x_{ij}^{v}y_{i}^{v}Z_{v}^{g}f_{ijgh}v_{ijgh}(t_{j}-t_{i}),$$ (9)

The power consumption cost $f_{2}^{\prime \prime }$ for electric vehicles is calculated as:

$$f_{2}^{\prime \prime }=k_{2}\sum_{g\in g_{3}}\sum_{v\in V}\sum_{j\in \{p\cup M\}}\sum_{i\in \{p\cup M\}}x_{ij}^{v}y_{j}^{v}Z_{v}^{g}l_{ij},$$ (10)

(3) Carbon Emission Cost *f*_3_:

$$f_{3}=k_{3}m_{{co}_{2}}=k_{3}\sum_{h\in u}\sum_{g\in \{g_{1},g_{2}\}}\sum_{v\in V}\sum_{j\in \{p\cup M\}}\sum_{i\in \{p\cup M\}}x_{ij}^{v}y_{j}^{v}Z_{v}^{g}\varepsilon_{ijgh}v_{ijgh}(t_{j}-t_{i}),$$ (11)

(4) Cooling Cost *f*_4_: During fresh product delivery, vehicles incur certain costs to maintain the temperature inside the compartments. As the compartments are opened during delivery, the unit cooling cost during delivery differs from that during non-delivery. The specific calculation method is as follows:

$$f_{4}=\sum_{h\in u}\sum_{g\in G}\sum_{j\in \{p\cup M\}}\sum_{j\in \{p\cup M\}}x_{ij}^{v}y_{j}^{v}Z_{v}^{g}(\frac{{fr}_{g}l_{ij}}{v_{ijgh}}+{fl}_{g}T_{j}),$$ (12)

(5) Freshness π Calculation

As per relevant reference [26], the freshness decay function for fresh products is $\theta (\tau ,t)=\frac{\tau^{\beta }}{1+{\alpha t}^{2}}$, where τ represents the preservation efforts level for freshness, α is the time sensitivity coefficient for freshness, β is the preservation effort sensitivity coefficient for freshness, and *π*_0_ represents the initial freshness of fresh products when leaving the depot. The average freshness at the time of fresh product delivery to all customers is calculated as:

$$\pi =\frac{\pi_{0}}{n}\sum_{i\in P}\frac{\tau^{\beta }}{1+\alpha t_{i}^{2}},$$ (13)

#### 3.2.2 Fresh delivery model

Objective Function

$$\min\left(F\right)=f_{1}+f_{2}+f_{3}+f_{4},$$ (14)

$$\min\left(m_{{co}_{2}}\right)=\sum_{h\in u}\sum_{g\in \{g_{1},g_{2}\}}\sum_{v\in V}\sum_{j\in \{p\cup M\}}\sum_{i\in \{p\cup M\}}x_{ij}^{v}y_{j}^{v}Z_{v}^{g}\varepsilon_{ijgh}v_{ijgh}(t_{j}-t_{i}),$$ (15)

$$\max\left(\pi \right)=\frac{\pi_{0}}{n}\sum_{i\in P}\frac{\tau^{\beta }}{1+\alpha t_{i}^{2}},$$ (16)

Constraints

$$\sum_{g\in G}\sum_{v\in V}\sum_{j\in P}x_{ij}^{v}y_{j}^{v}Z_{v}^{g}=1,i\in \{P\cup M\},$$ (17)

$$\sum_{m\in M}\sum_{i\in P}x_{mi}^{v}y_{i}^{v}=\sum_{m\in M}\sum_{i\in P}x_{im}^{v}y_{i}^{v},v\in V,$$ (18)

$$\sum_{i\in P}x_{im}^{v}y_{i}^{v}+\sum_{i\in P}x_{mi}^{v}y_{i}^{v}>1,v\in V,m\in M,$$ (19)

$$l_{mi}x_{im}^{v}\leq R_{m},m\in M,v\in V,i\in P,$$ (20)

$$t_{i}\leq {tti}_{i},i\in P,$$ (21)

$$t_{j}=\left\{ \begin{array}{c} \sum_{h\in u}\sum_{g\in G}\sum_{v\in V}\sum_{j\in \{P\cup M\}}\sum_{i\in P}(\frac{l_{ij}x_{ij}^{v}y_{j}^{v}Z_{v}^{g}}{v_{ijgh}}+{Ti}_{i}),\\ \sum_{h\in u}\sum_{g\in G}\sum_{v\in V}\sum_{j\in P}\sum_{i\in M}(\frac{l_{ij}x_{ij}^{v}y_{j}^{v}Z_{v}^{g}}{v_{ijgh}}+bt), \end{array}\right.$$ (22)

$$\sum_{h\in u}\sum_{v\in V}\sum_{j\in \{P\cup M\}}\sum_{i\in \{P\cup M\}}x_{ij}^{v}y_{j}^{v}Z_{v}^{g}{load}_{v}^{h}\leq m_{g},g\in G,$$ (23)

$$\sum_{j\in \{P\cup M\}}\sum_{i\in \{P\cup M\}}x_{ij}^{v}y_{j}^{v}Z_{v}^{g}l_{ij}\leq L_{g},g\in G,v\in V,$$ (24)

$$\frac{\tau^{\beta }}{1+\alpha t_{i}^{2}}\geq \bar{\pi },$$ (25)

$$x_{ij}^{v}=\{0,1\},$$ (26)

$$y_{j}^{v}=\{0,1\},$$ (27)

$$Z_{v}^{g}=\{0,1\},$$ (28)

Eq (14) represents the minimum cost; Eq (15) represents the minimum carbon emissions; Eq (16) represents the maximum freshness; Eq (17) indicates that each customer point can only be serviced by a vehicle once; Constraint (18) indicates that the number of departures from and returns to the depot are the same for each vehicle; Constraint (19) indicates that vehicle v replenishes at depot m; Eq (20) indicates that customers served by depot m must be within its service radius; Eq (21) indicates that the arrival time at customer points must not exceed the latest time; Eq (22) denote the continuity of time, where Eq (21) denotes the previous node being a customer, otherwise Eq (22); Eq (23) indicates that the real-time load of the vehicle is less than or equal to the maximum load; Eq (24) indicates that the distance traveled by the vehicle must not exceed its maximum distance; Eq (25) expresses that the freshness at the customer point upon arrival must not be lower than the minimum freshness; Eq (26) specifies that vehicle v travels from node i to j (i≠j); Eq (27) denotes that the vehicle v is delivering to node j; Eq (28) indicates the vehicle v’s type as g.

## 4 Solution algorithm

The model developed in this paper is a multi-objective optimization model. Commonly in academia, one of the objective functions is set to a minimum threshold, which is then converted into a constraint, thus transforming the multi-objective optimization model into a single-objective optimization model. These two methods are simple to implement, but the setting of threshold conditions is subjective and the algorithm typically results in only one feasible solution. The NSGA-II algorithm, introduced in 2002 by Gopal [27], is a second-generation version based on the NSGA. It uses a fast non-dominant sorting strategy and a crowding distance operator during iterations, providing a Pareto optimal set of solutions in each iteration. This allows businesses to choose from a more diverse set of distribution options, suitable for solving multi-objective optimization models, and has been widely used by scholars in the last decade to solve logistics distribution models [28, 29] and other related optimization problems [30]. Although the NSGA-II method has certain advantages in solving multi-objective optimization problems, it has limited local search capabilities and slow convergence, particularly in problems with large solution spaces, often getting stuck in local optima in the mid to late iterations.

The Large Neighborhood Search (LNS) can search the neighborhood of feasible solutions to find the optimal solution and has strong local search capabilities. Therefore, we embedded the Large Neighborhood Search into the traditional NSGA-II algorithm to create a hybrid algorithm (LNSNSGA-II). Its basic idea is to optimize the chromosome after each crossover and mutation operation using Large Neighborhood Search, then perform fast non-dominant sorting and crowding distance calculation on the population. Chromosomes in the top tier are retained as part of the Pareto optimal set for the next iteration’s selection, allowing iteration-based achievement of the Pareto optimal set based on LNSNSGA-II.

### 4.1 Chromosome processing

#### 4.1.1 Initial chromosome definition

Chromosomes are generated using a construction method. First, the order of the customer numbers to be served and the vehicle numbers are shuffled, resulting in {k_points} and {v_points}. Then, each customer number is successively inserted into each vehicle number in {v_points} according to the following principles:

Step 1: Determine the vehicle’s affiliated depot M, maximum load m_max, maximum travel distance l_max, minimum freshness π¯, and set l = 0, t = 0, m = 0.

Step 2: Each customer point in {k_points} is successively inserted into the vehicle after being checked for the current traffic conditions based on t. The vehicle’s travel speed is calculated, as well as the distance dis from the customer point to the depot. After inserting the customer point, calculate the current load m’, travel distance l’, arrival time t’, the latest arrival time tti’, and the freshness π’ upon arrival at the customer point. If the conditions m’< = m_max, l’+dis< = L_max, t’< = tti’, π’> = π¯ are not met, proceed to Step 3; otherwise, insert the customer point into the delivery orders of the current vehicle, remove the customer point from {k_points}, and update t = t’, m = m’, l = l’. If {k_points} is empty, proceed to Step 5.

Step 3: Determine if a restocking strategy is to be used. If only m’ does not meet the conditions, then a restocking strategy is adopted, leading to Step 4; otherwise, proceed to Step 2 to assign the next customer point to be served to the vehicle in {v_points}.

Step 4: Identify the depot M’ closest to the previous customer point, and insert depot M’ after the previous customer point. Then, successively take out the unassigned customer points from {k_points}, and go back to Step 2 while updating l = 0, m = 0.

Step 5: Complete the vehicle delivery arrangements for all customer points, thus constructing the chromosome.

#### 4.1.2 Large neighborhood search design

To accommodate the unique chromosome construction in this paper, the large neighborhood search algorithm has been enhanced to better explore the solution space. The specific search steps are as follows:

Step 1: Decode the chromosome into v_num sub-chromosomes, where v_num represents the number of vehicles. Conduct a neighborhood search on each sub-chromosome (representing the order of vehicles delivering customers) in sequence.

Step 2: Determine whether this sub-chromosome involves restocking along the way. If not, conduct a neighborhood search on the customer points delivered by the vehicle. Under the condition that the customer point arrival times are met, find a new sub-chromosome with the shortest vehicle travel distance to replace the old sub-chromosome. If restocking is involved, proceed to Step 3.

**Fig 1:**
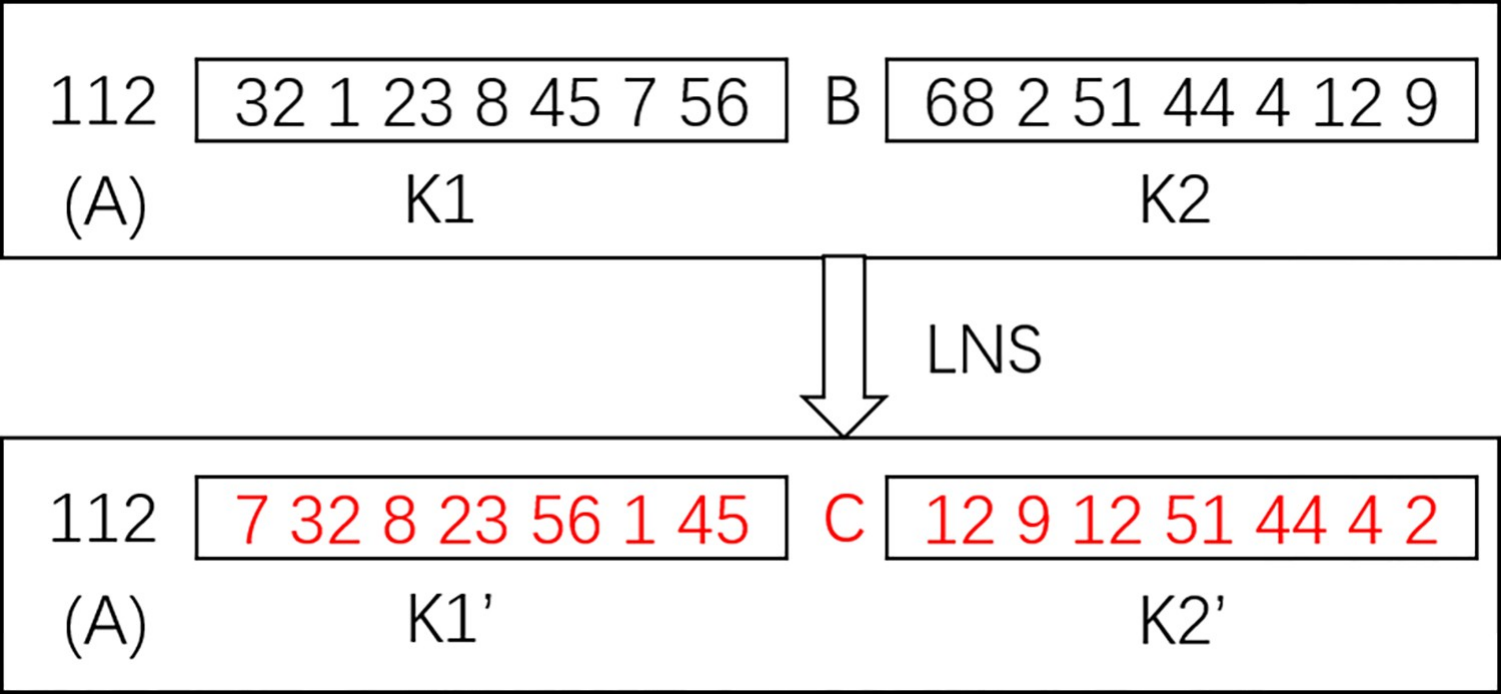
Diagram of sub-chromosomal renewal.

Step 3: Identify the location of restocking in the sub-chromosome. Conduct a neighborhood search on the customer set {K1} before restocking. Under the condition that the customer point arrival times are met, adjust and rearrange the delivery order of these customers into {K1’} with the shortest vehicle travel distance as the target, and then insert the depot closest to the last customer in {K1’}; then, conduct a neighborhood search to change {K2} after restocking into {K2’}. As shown in Fig 1, the sub-chromosome, where 112 represents the vehicle number belonging to depot A, with A, B, and C representing depot numbers, {K1} and {K2} are transformed into {K1’} and {K2’} through a neighborhood search, while the depot for restocking changes from B to C. Replace the old sub-chromosome with the modified sub-chromosome.

Step 4: Conduct neighborhood search using Step 2 and Step 3 on all sub-chromosomes sequentially until all sub-chromosomes have completed the search, ending the search process.

### 4.2 LNSNSGA-II process description

Algorithm steps are as follows:

Step 1: Population initialization. Initialize various variables and parameters, including the population size N, iteration count iter set to 1, maximum iteration count MaxIt, crossover probability pc, and mutation probability pm.

Step 2: Calculate the multi-objective values and perform non-dominated sorting and crowding distance calculation on the initial population based on the size of the objective values, selecting Q individuals in the first level.

Step 3: Elite selection strategy selects N individuals from the N individuals.

Step 4: Perform crossover and mutation strategy on the above N individuals to obtain N individuals.

Step 5: Perform large neighborhood search strategy on the N individuals to obtain R individuals.

Step 6: Merge the population to obtain N+R+Q individuals.

Step 7: Calculate the multi-objective values and perform non-dominated sorting and crowding distance calculation on the population based on the size of the objective values, selecting Q individuals in the first level.

Step 8: Select N individuals to form the next generation population according to the elite selection strategy.

Step 9: iter = iter+1, if iter< = MaxIt, go to Step 4;

Step 10: Output the Pareto optimal solution set, and end the algorithm.

**Fig 2:**
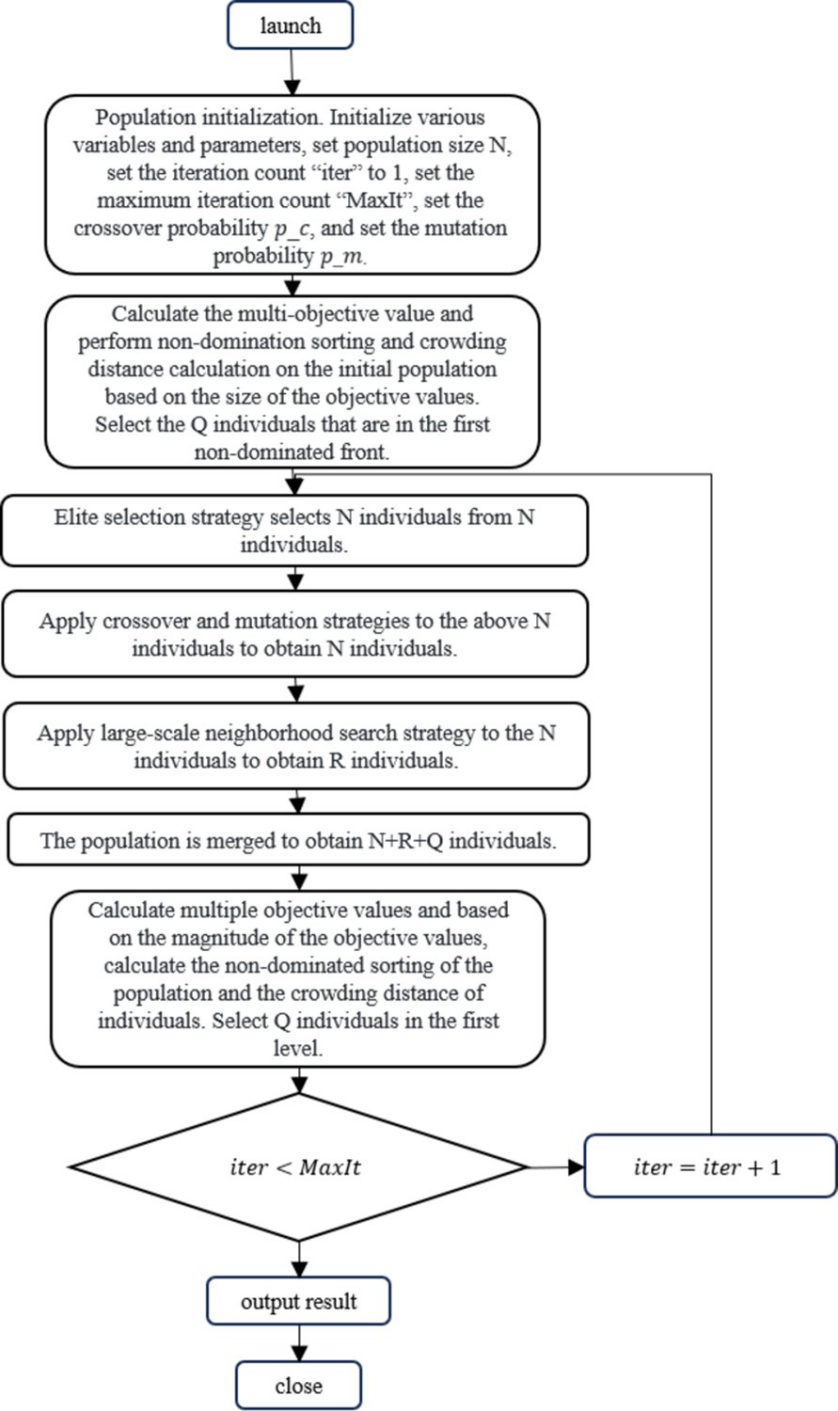
Flowchart of the LNSNSGA-II algorithm.

The algorithm flowchart is shown in Fig 2:

## 5 Results and discussion

The coordinates of customer points, demand, latest delivery time, service time, and other data for the case study in this paper can be found in the S1 Table. There are a total of 100 customer points to be served in the case study. The information for the depot and vehicles is shown in Table 2. Vehicle types *g*_1_ and *g*_2_ represent gasoline vehicles, whose speed is affected by traffic conditions. Vehicle type *g*_3_ represents electric vehicles, whose speed is not affected by traffic conditions.

**Table 2:** Vehicle and depot information.

Vehicle ID	Depot ID	Depot Coordinates	Vehicle Type	Vehicle ID	Depot ID	Depot Coordinates	Vehicle Type
101	Center1(A)	(47,45)	*g*_1_	116	Center2(B)	(38,10)	*g*_2_
102	Center1(A)	(47,45)	*g*_2_	117	Center2(B)	(38,10)	*g*_1_
103	Center1(A)	(47,45)	*g*_1_	118	Center2(B)	(38,10)	*g*_2_
104	Center1(A)	(47,45)	*g*_2_	119	Center2(B)	(38,10)	*g*_2_
105	Center1(A)	(47,45)	*g*_3_	120	Center2(B)	(38,10)	*g*_2_
106	Center1(A)	(47,45)	*g*_1_	121	Center3(C)	(20,43)	*g*_3_
107	Center1(A)	(47,45)	*g*_3_	122	Center3(C)	(20,43)	*g*_1_
108	Center1(A)	(47,45)	*g*_2_	123	Center3(C)	(20,43)	*g*_1_
109	Center1(A)	(47,45)	*g*_1_	124	Center3(C)	(20,43)	*g*_2_
110	Center1(A)	(47,45)	*g*_3_	125	Center3(C)	(20,43)	*g*_3_
111	Center2(B)	(38,10)	*g*_3_	126	Center3(C)	(20,43)	*g*_1_
112	Center2(B)	(38,10)	*g*_1_	127	Center3(C)	(20,43)	*g*_2_
113	Center2(B)	(38,10)	*g*_2_	128	Center3(C)	(20,43)	*g*_1_
114	Center2(B)	(38,10)	*g*_3_	129	Center3(C)	(20,43)	*g*_3_
115	Center2(B)	(38,10)	*g*_1_	130	Center3(C)	(20,43)	*g*_3_

To meet the testing requirements, the following additional data is provided:

(1) Assume that the distribution center operates 24 hours a day, starting distribution at 6:00 in the morning.

(2) The time interval tu for calculating the traffic congestion index is set to 15 minutes.

(3) According to the traffic rules in major cities, this study sets 7:00–8:00, 12:00–13:00, and 16:00–17:00 as peak traffic congestion periods (traffic congestion index greater than 8). The remaining time periods are set as non-severe congestion periods. The traffic congestion index values for the 96 time intervals are calculated using Eq (1) and are as follows: *z*_*h*_ = [0,6,2,2,9,10,10,10,9,9,9,10,1,6,0,1,6,0,7,5,7,7,6,3,10,9,9,10,10,10,9,10,0,5,3,0,0,7,2,0,2,1,0,0,9,9,10,10,9,10,10,9,4,5,3,4,6,2,6,2,1,7,6,1,4,1,4,0,4,5,0,7,6,3,1,2,0,4,7,7,6,5,2,2,3,3,2,3,7,3,2,3,6,7,0,4].

The algorithm is implemented using Matlab R2016b and run on a computer with an 11th Gen Intel® Core™ i7-1165G7 @ 2.80GHz processor and 16.0 GB of RAM. The program is set with the following parameters: *m*_1_ = 150*kg*, *m*_2_ = 90*kg*; *m*_3_ = 30*kg*, *v*_1_ = 50*km*/*h*; *v*_2_ = 60*km*/*h*, *v*_3_ = 30*km*/*h*, *L*_1_ = 300*km*, *L*_2_ = 120*km*, *L*_3_ = 50*km*, *c*_1_ = 300*yuan*/*each*, *c*_2_ = 200*yuan*/*each*, *c*_3_ = 100*yuan*/*each*, *k*_1_ = 0.8*yuan*/*km*, *k*_2_ = 0.1528*yuan*/*kg*, *k*_3_ = 7.5*yuan*/*liters*, *fr*_1_ = 5*yuan*/*h*, *fr*_2_ = 3.5*yuan*/*h*, *fr*_3_ = 2*yuan*/*h*, *fl*_1_ = 5.3*yuan*/*h*, *fl*_2_ = 4*yuan*/*h*, *fl*_3_ = 2.5*yuan*/*h*, *R* = 36*km*, *φ*_0−6_ = (110,0,0,0.000375,8702,0,0), *β*_0−7_ = (1.27,0.0614,0,−0.0011,−0.00235,0,0,−1.33), *π*_0_ = 1; *τ* = 0.8, *α* = 0.1, *β* = 0.15.

### 5.1 Algorithm comparative analysis and discussion

Based on our experimental instances and model, we conducted 10 simulation experiments using both the pre-improved multi-objective genetic algorithm and the post-improved multi-objective genetic algorithm (LNSNSGA-II). Each NSGA-II algorithm run would generate a set of Pareto solutions, with the Pareto frontier representing the optimal solution set obtained in each run. The solutions in the set are non-dominated. The nature of multi-objective optimization algorithms dictates that the solutions generated cannot obtain all optimal values. Experimental data generated by each operation is shown in Table 3. *x*_*i*_ represents the lowest total logistics distribution cost in the Pareto solution set, *x*_*j*_ represents the average total logistics distribution cost. *y*_*i*_ represents the vehicle carbon emissions when obtaining the lowest total logistics distribution cost, *y*_*j*_ represents the average vehicle carbon emissions in the Pareto solution set. *z*_*i*_ represents the freshness of goods when obtaining the lowest total logistics distribution cost, *z*_*j*_ represents the average freshness of goods in the Pareto solution set.

**Table 3:** Comparison of algorithm execution results.

Number of runs	LNSNSGA-Ⅱ						NSGA-Ⅱ					
	*x*_*i*_	*y*_*i*_	*z*_*i*_	*x*_*j*_	*y*_*j*_	*z*_*j*_	$x_{i}^{\prime }$	$y_{i}^{\prime }$	$z_{i}^{\prime }$	$x_{j}^{\prime }$	$y_{j}^{\prime }$	$z_{j}^{\prime }$
**1**	10201.4	888.7	0.8658	11215.8	1091.4	0.8488	11292.3	1108.3	0.8543	12055.5	1168.0	0.8356
**2**	10105.8	894.9	0.8661	11422.2	1075.6	0.8492	10815.2	1022.5	0.8478	11862.8	1108.7	0.8425
**3**	9859.7	892.1	0.8655	11322.3	1072.5	0.8442	11520.8	1203.8	0.8522	11759.8	1190.3	0.8341
**4**	10112.3	921.3	0.8567	11492.1	1034.6	0.8504	11332.7	1134.2	0.8653	11819.3	1213.2	0.8388
**5**	10221.8	900.5	0.8621	11832.9	1105.2	0.8511	12072.2	1047.2	0.8564	12101.9	1156.2	0.8373
**6**	9987.2	872.3	0.8533	11205.0	1058.7	0.8468	10921.4	1038.2	0.8521	11672.1	1176.8	0.8412
**7**	10007.1	888.2	0.8701	11502.2	1065.3	0.8487	11291.6	1124.5	0.8521	11859.2	1202.4	0.8367
**8**	10170.6	902.1	0.8632	10893.2	1088.8	0.8523	11382.1	1146.2	0.8489	12328.7	1178.2	0.8401
**9**	9779.8	896.2	0.8611	10949.5	1029.4	0.8521	11528.1	1183.6	0.8582	12288.4	1188.8	0.8387
**10**	10201.5	872.4	0.8671	11608.3	1045.6	0.8492	10988.4	1135.9	0.8544	11578.3	1202.5	0.8366

To demonstrate the superiority of our proposed improved multi-objective non-dominated sorting genetic algorithm (LNSNSGA-II), a t-test based on the MATLAB platform was used for significance testing. As the overall mean and variance are unknown, the Shapiro-Wilk test was first used to verify the normal distribution characteristics of the sample data, with the following hypotheses:

H0: The sample follows a normal distribution.

H1: The sample does not follow a normal distribution.

When the confidence level is 0.05, if h = 0 and p ≥ 0.05, the null hypothesis is accepted; if h = 1 and p < 0.05, the null hypothesis is rejected. Test results from Table 4 show that each set of data follows a normal distribution and can undergo a t-test for significance.

**Table 4:** Shapiro-Wilk test results.

		LNSNSGA-Ⅱ					NSGA-Ⅱ						
Sample Size		10					10						
**Objective Function Value**		***x***_***i***_	***y***_***i***_	***z***_***i***_	***x***_***j***_	***y***_***j***_	***z***_***j***_	$x_{i}^{\prime }$	$y_{i}^{\prime }$	$z_{i}^{\prime }$	$x_{j}^{\prime }$	$y_{j}^{\prime }$	$z_{j}^{\prime }$
**Regular Parameters**	**Standard Deviation**	10064.7	892.9	0.8631	11344.3	1066.7	0.8493	11314.5	1114.4	0.8542	11932.6	1178.5	0.8382
		152.7	14.3	0.005	290.0	25.0	0.0025	361.8	61.06	0.005	252.5	30.0	0.0026
**h**		0	0	0	0	0	0	0	0	0	0	0	0
**p**		0.27	0.50	0.43	0.50	0.50	0.26	0.49	0.50	0.44	0.25	0.49	0.50

After verifying the normal distribution characteristics of the sample data, a t-test was performed for each set of data with the following hypotheses:

H0: The average difference in the same type of objective function values under the two algorithms is zero.

H1: The average difference in the same type of objective function values under the two algorithms is not zero.

Set the confidence level at 0.05. When h = 1 and p < 0.05, the null hypothesis H0 does not hold, and the difference is significant; otherwise, H0 is accepted. A t-test was conducted on four pairs of sample data, with corresponding test results as shown in Table 5.

**Table 5:** t-test analysis results.

	$\left(x_{i},x_{i}^{\prime }\right)$	$\left(y_{i},y_{i}^{\prime }\right)$	$\left(z_{i},z_{i}^{\prime }\right)$	$\left(x_{j},x_{j}^{\prime }\right)$	$\left(y_{j},y_{j}^{\prime }\right)$	$\left(z_{j},z_{j}^{\prime }\right)$
h	1	1	1	1	1	1
p	3.1041*e*^−7^	5.7642*e*^−7^	0.000895	0.000139	5.2052*e*^−8^	1.1912*e*^−8^

From Table 5, it can be seen through the t-test analysis that there are significant differences in the lowest total logistics distribution costs, lowest total vehicle carbon emissions, and highest freshness of goods. The hypothesis H1 was accepted, indicating significant differences in the average values of the lowest logistics distribution costs, lowest total vehicle carbon emissions, and highest freshness of goods before and after the algorithm improvements.

By comparing their average values, it can be concluded that the improved algorithm reduced the lowest total logistics distribution cost by 11.1%, the average total carbon emissions of vehicles by 9.5%, and increased the average freshness of goods by 1.1%. Although the improvement in the freshness of goods was not significant, the lowest total vehicle carbon emissions were reduced by 19.9%. Additionally, the runtime for 300 iterations of the algorithm before improvement was about 20 minutes, but after improvement, it increased to about 35 minutes due to the enhanced local search capability of the improved algorithm, thus increasing the runtime. However, the runtime is within a reasonable range.

**Fig 3:**
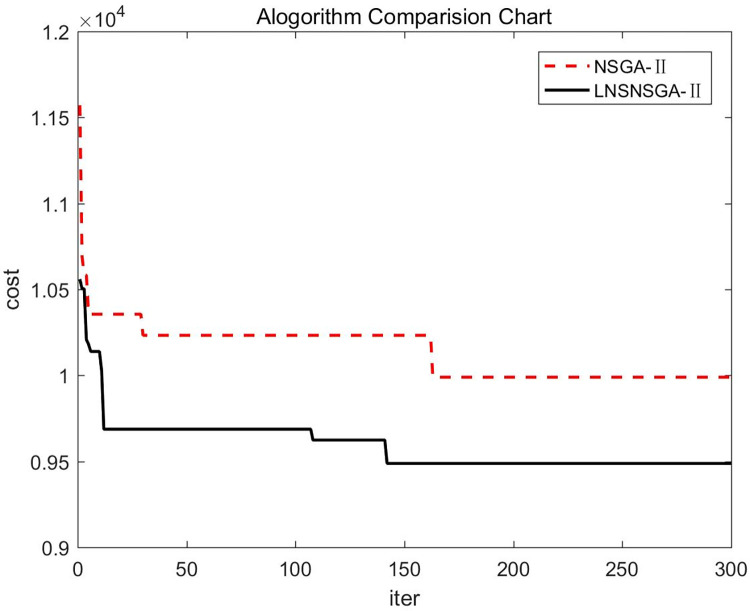
Comparison of algorithms.

To compare the convergence differences before and after the algorithm improvements, the optimal solution with the lowest total logistics distribution cost was selected from 10 simulation experiments, and the convergence curves were compared as shown in Fig 3.

The comparative analysis through Fig 3 revealed that our designed improved algorithm performs better in addressing cold chain logistics distribution problems considering traffic congestion and en route restocking than the traditional NSGA-II algorithm. The improved LNSNSGA-II algorithm is capable of finding high-probability sub-optimal solutions in the early to mid-phases, with strong search capabilities, while the traditional NSGA-II algorithm gets stuck in local optima and struggles to break out in the later phases.

### 5.2 Comparative analysis and discussion on different numbers of vehicle depots

Additionally, we also studied the impact of different numbers of vehicle depots on distribution costs and vehicle carbon emissions. We conducted experimental verifications with single-depot (1-center) distribution, dual-depot (2-center) distribution, and triple-depot (3-center) distribution. The single depot was positioned at coordinates (35,35) with a service radius of 50km, and the dual depots were located at (18,40) and (52,40) respectively, each with a service radius of 40km.

**Fig 4:**
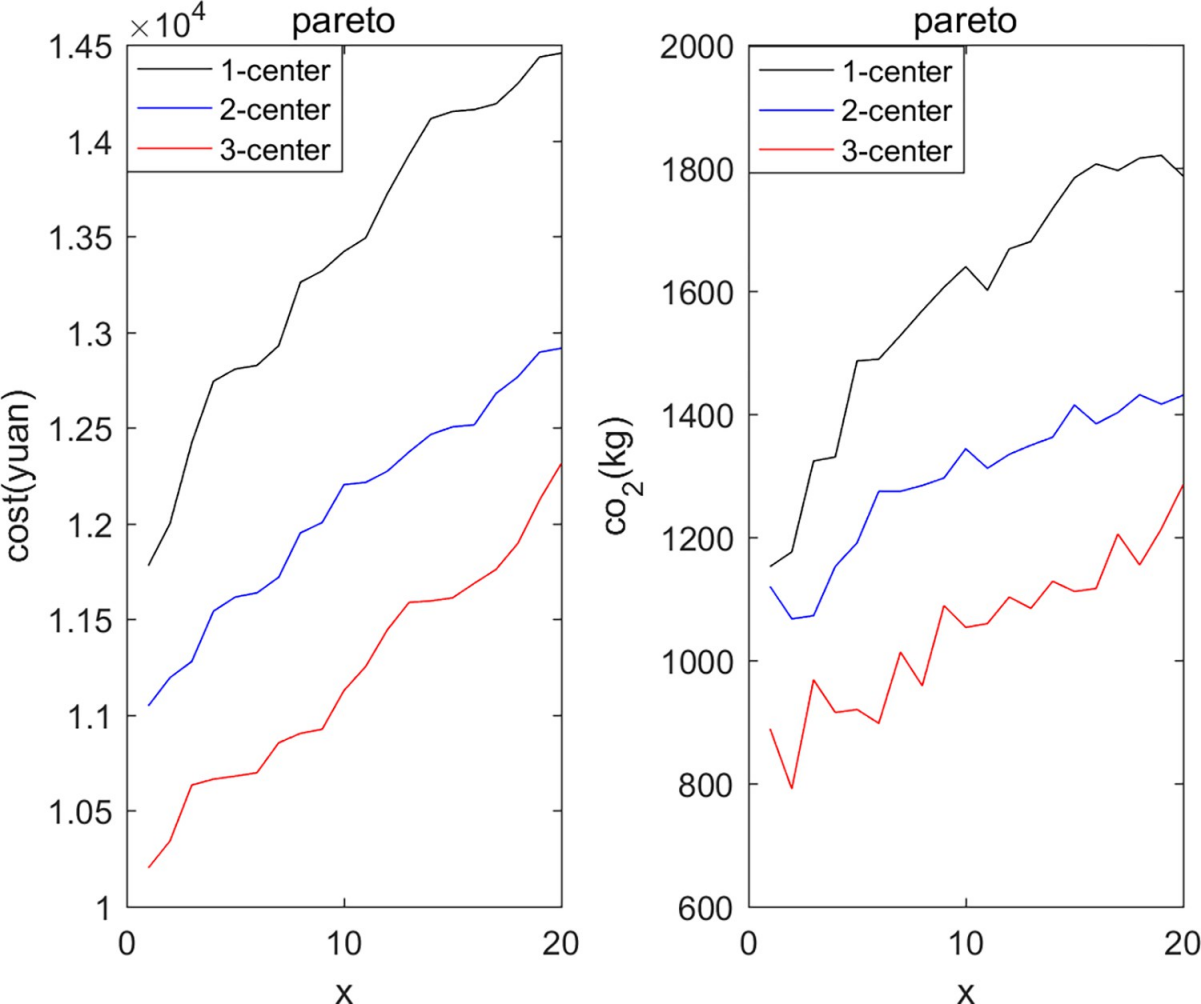
Comparison of results for multiple car parks.

By setting the number of delivery depots to be one, two, and three respectively, the multi-objective planning solutions obtained from Pareto-optimality are presented in Fig 4. Fig 4 (left) shows the Pareto-optimal solutions for cost, and Fig 4 (right) shows the Pareto-optimal solutions for carbon emissions. It can be observed that, as the number of depots increases, both delivery costs and vehicle carbon emissions are reduced to a certain extent. This is because this study considers the delivery strategy of replenishment along the way, allowing vehicles to replenish at nearby depots during the delivery process.

**Fig 5:**
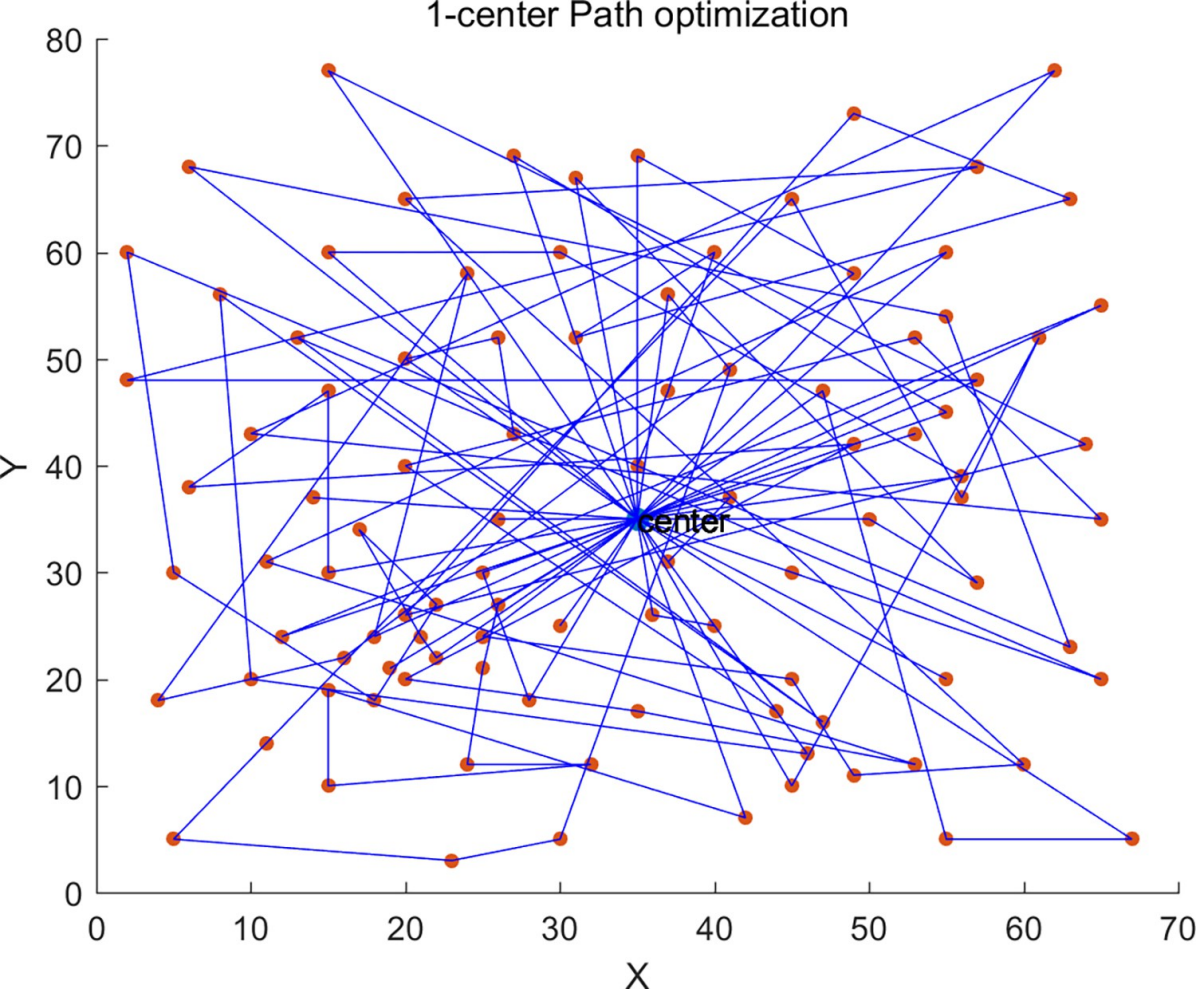
1-center delivery scheme.

**Fig 6:**
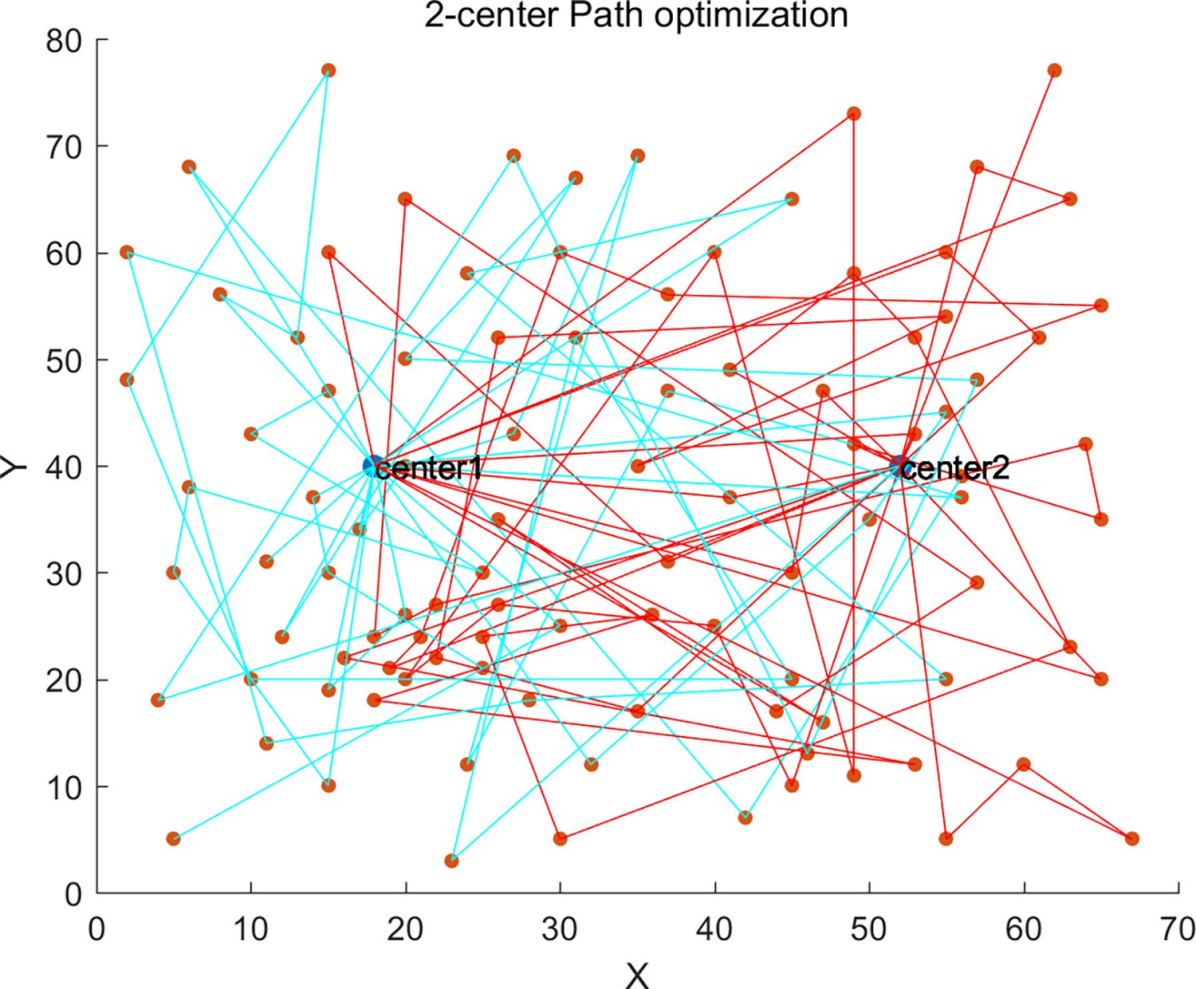
2-center delivery scheme.

**Fig 7:**
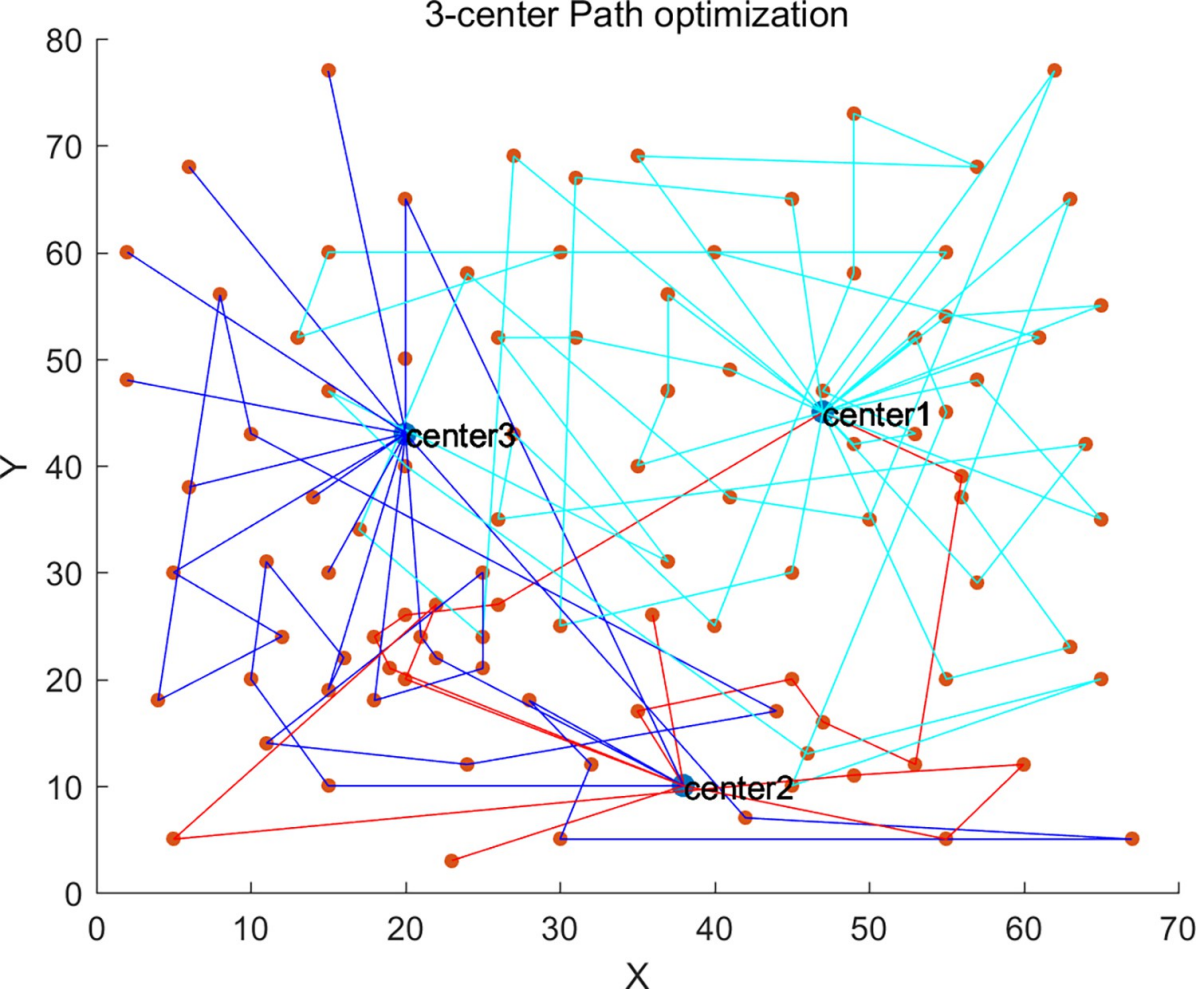
3-center delivery scheme.

The path planning diagrams for single-depot, dual-depot, and triple-depot are shown in Figs 5–7 respectively. It can be observed that as the number of depots increases, vehicles will replenish at other depots along the way, apart from the starting depot. This strategy effectively reduces the vehicles’ unnecessary travel distance, thereby lowering the costs and carbon emissions generated during the delivery process.

The results of vehicle path planning for the triple-depot with replenishment strategy are presented in Table 6, where 16 vehicles are found to be involved in the delivery tasks. Among them, vehicles 101, 104, 109, 114, 120, 126, 129, and 130 engage in replenishment along the way, fully leveraging the delivery performance of the vehicles.

**Table 6:** Path planning results.

No.	routes	No.	routes
**101**	A-9-19-48-10-30-78-A-33-3-22-25-74-18-82-53-88-31-1-A	**116**	——
**102**	A-24-79-A	**117**	——
**103**	——	**118**	B-98-93-99-94-A-68-56-72-21-2-B
**104**	A-81-34-A-35-80-55-4-A	**119**	——
**105**	——	**120**	B-41-67-15-57-87-B-14-16-84-85-91-B
**106**	——	**121**	C-45-C
**107**	A-70-69-27-A	**122**	——
**108**	A-20-90-13-26-A	**123**	——
**109**	A-32-71-66-51-40-52-89-29-54-A-63-95-60-62-28-12-65-50-77-76-A	**124**	C-64-C
**110**	——	**125**	C-36-C-83-C
**111**	——	**126**	C-49-C-17-61-86-47-8-73-42-44-6-97-100-C
**112**	B-37-96-38-75-39-23-B	**127**	——
**113**	——	**128**	——
**114**	B-43-B-58-B	**129**	C-46-C-5-C
**115**	——	**130**	C-7-11-B-92-59-C

### 5.3 Analysis of simulated random traffic congestion index

In this section, we tested the impact of random traffic conditions on our model and algorithm. We generated random traffic congestion indices for non-peak traffic periods to represent simulated road traffic conditions. For the peak traffic periods, we similarly generated random indices representing high congestion levels to simulate road traffic conditions. We also considered a logistics distribution model with three depots and cross-depot en-route restocking, solving it using the LNSNSGA-II algorithm.

The results of running LNSNSGA-II five times are shown in Table 7. From Table 7, it is evident that the fluctuation range for the lowest total logistics distribution cost (cost) was [-1.6%, 1.53%]. At the same time, the total carbon emissions (co2) and freshness of goods (fre) under the lowest total logistics cost showed minimal fluctuation, indicating that our algorithm’s solution stability is strong under random traffic conditions.

**Table 7:** Path planning results for stochastic traffic conditions.

No.	cost	Co2	fre
**1**	10201.4	888.7	0.8672
**2**	10287.5	872.3	0.8633
**3**	10023.1	917.2	0.8601
**4**	9970.6	902.4	0.8632
**5**	10179.8	896.5	0.8611
**Mean**	10132.5	895.4	0.8630
**min**	9970.6	872.3	0.8601
**max**	10287.5	917.2	0.8672

Furthermore, to study the impact of the traffic update interval (tu) on the lowest total logistics distribution cost, vehicle carbon emissions, and freshness of goods, we simulated random traffic conditions with tu intervals of 5 minutes, 10 minutes, 15 minutes, 20 minutes, and 25 minutes. The results obtained from the model are shown in Table 8. According to Table 8, both cost and co2 decrease as tu increases, while fre increases. This is because as tu increases, meaning that the change in road traffic conditions shifts from rapid to slow, the impact of traffic congestion on the speed of delivery vehicles decreases. Thus, the total delivery cost and total carbon emissions decrease, and the arrival time of goods at customer points is slightly advanced, resulting in an increase in the average freshness of the goods when they reach the customer.

**Table 8:** Analysis of planning results for different tu.

tu	cost	Co2	fre
**5**	10521.6	926.6	0.8427
**10**	10346.8	914.8	0.8568
**15**	10201.4	888.7	0.8624
**20**	10122.3	876.5	0.8698
**25**	9987.8	854.6	0.8721

### 5.4 Analysis of on-the-way replenishment strategy

**Fig 8:**
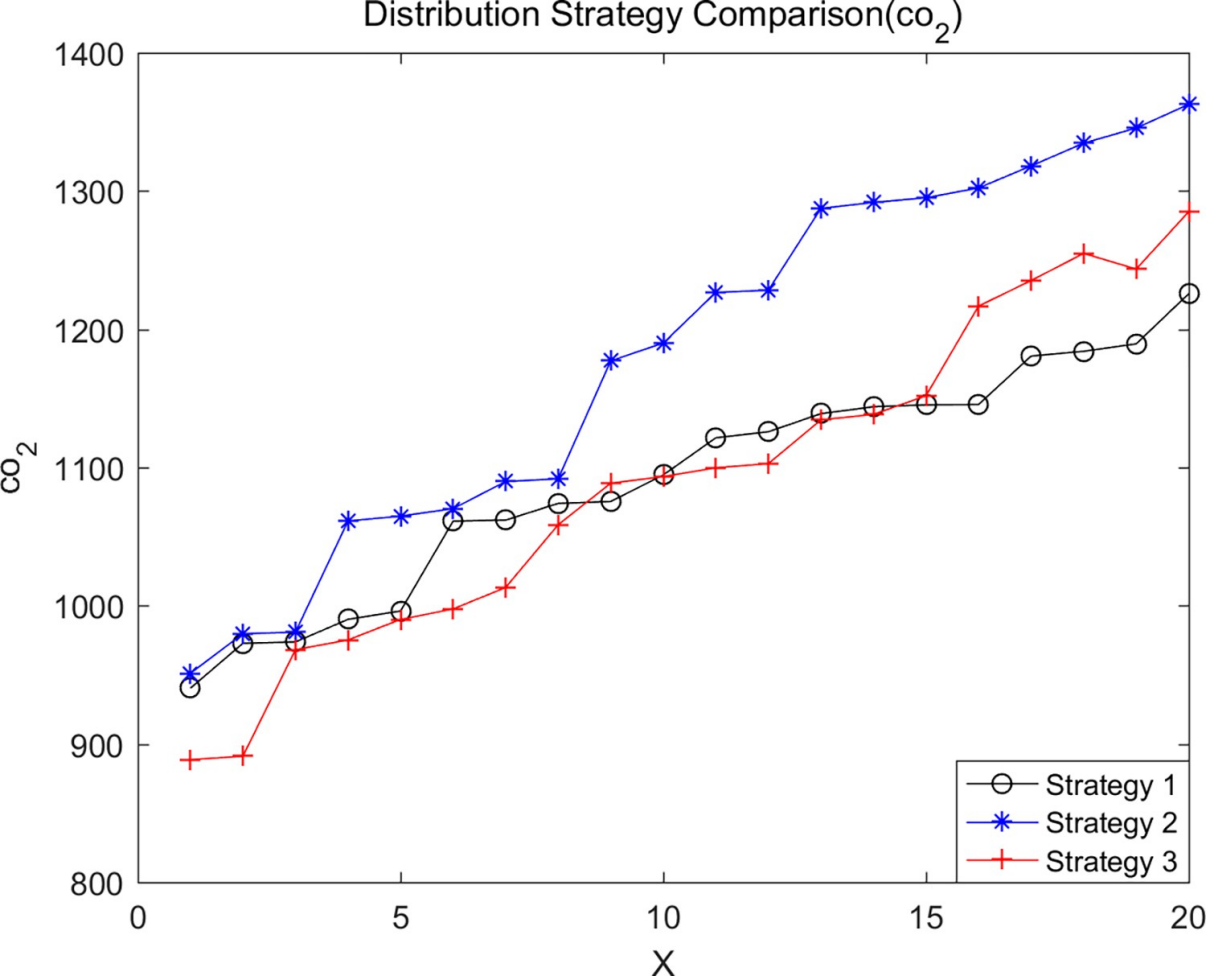
Total delivery cost comparison.

**Fig 9:**
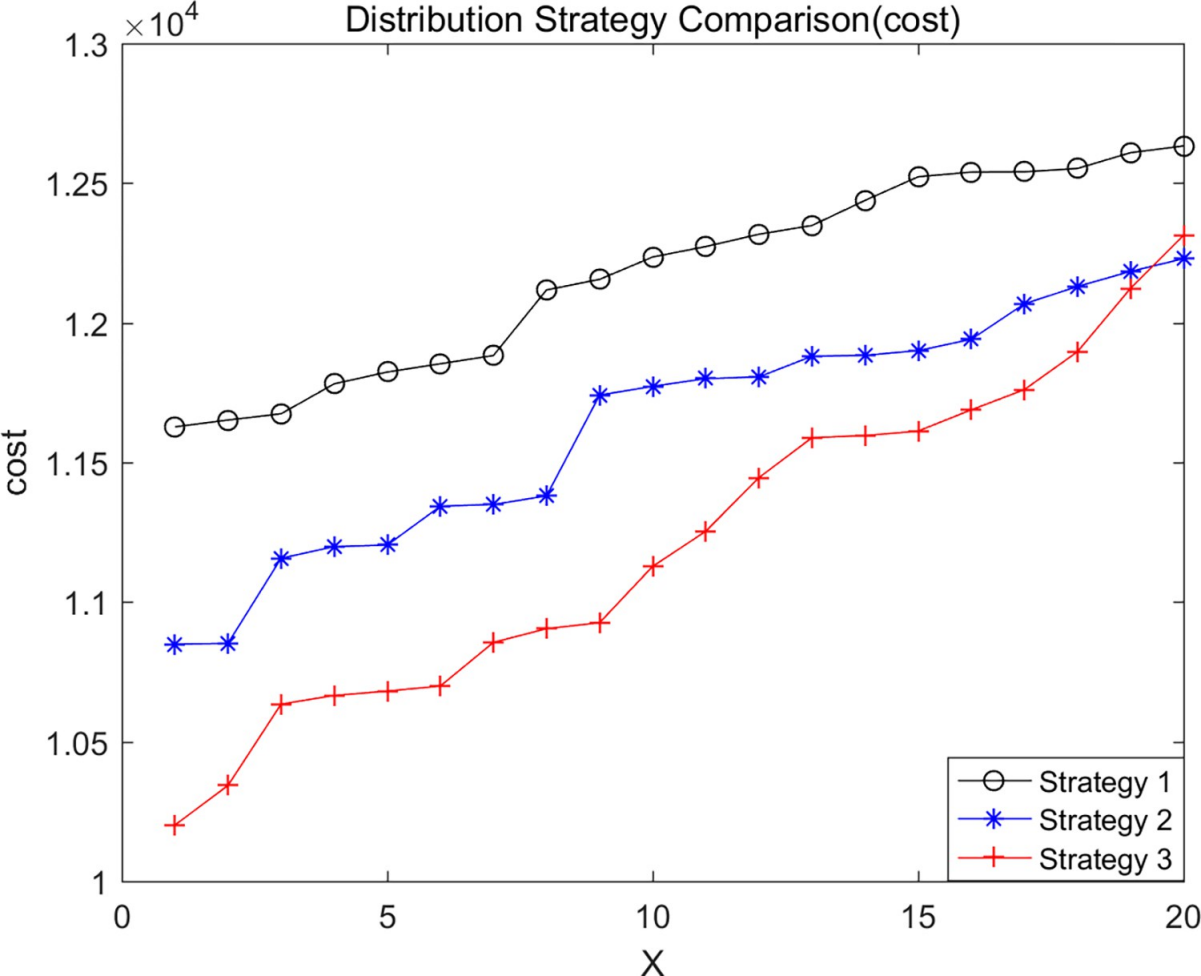
Comparison of total vehicle carbon emissions.

**Fig 10:**
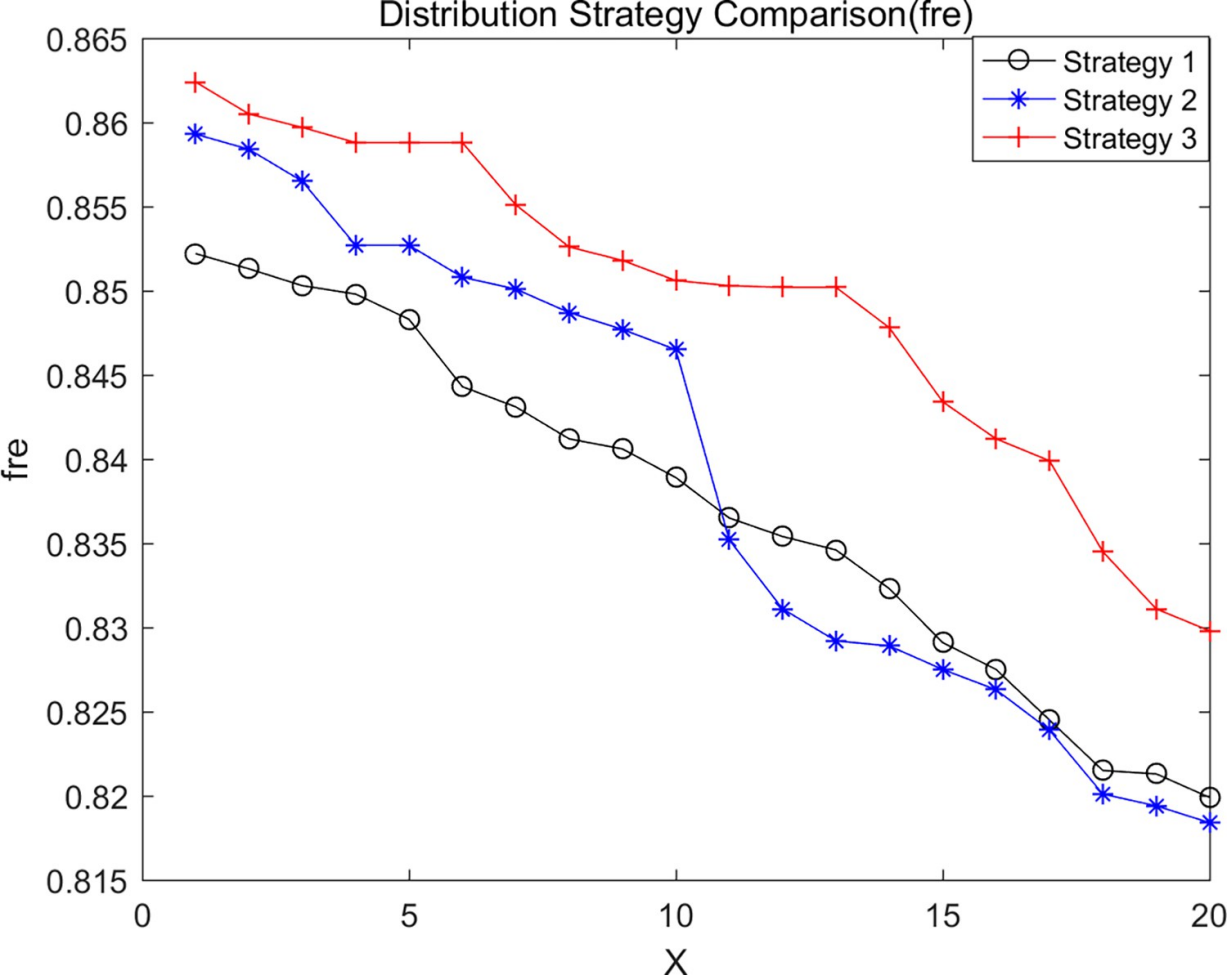
Comparison of freshness of good.

We analyzed distribution strategies by considering three different approaches: no en-route restocking (Strategy 1), en-route restocking without crossing depots (Strategy 2), and en-route restocking with the ability to cross depots (Strategy 3). These strategies were evaluated to understand their impact on the experimental results. Keeping specific parameters constant, we used LNSNSGA-II to run simulations with cost, co2, and fre as dominant objectives, resulting in the Pareto optimal solution sets shown in Figs 8–10.

The graphs indicate that in terms of minimizing total logistics distribution costs and vehicle carbon emissions while maximizing goods freshness, Strategy 3 performs the best, followed by Strategy 2, with Strategy 1 being the least effective. This suggests that considering the strategy of en-route restocking with the ability to cross depots offers certain advantages.

### 5.5 Analysis of parameters α, β, and τ

To verify the impact of ***α*, *β***, and ***τ*** on the freshness of goods, we set the freshness effort sensitivity factor (***β*** = 0.15) and conducted a sensitivity analysis on the time sensitivity factor ***α*** and the freshness effort level coefficient ***τ***.

As shown in Table 9, the average freshness of goods delivered to customer points increases with increases in ***α*** and ***τ***. This is because raising the level of freshness effort and reducing the time sensitivity factor can delay the impact of time on the freshness of perishables. This can enlighten enterprises to consider the type of goods being distributed before planning deliveries, to determine their sensitivity to time changes, and to appropriately enhance the effort to preserve the freshness of the goods.

**Table 9:** Analysis of freshness-related parameters.

*α*	*β*	*τ*	fre
0.01	0.15	0.125	0.73
0.03	0.15	0.25	0.81
0.05	0.15	0.375	0.86
0.08	0.15	0.75	0.95
0.1	0.15	0.8	0.96

## 6 Conclusion

Road traffic conditions are dynamic and difficult to accurately predict, posing a significant challenge to urban logistics distribution. In this study, we focused on this problem by simulating road traffic conditions (congestion index) and considering cold chain logistics as the main research subject. We also incorporated multi-depot and multi-vehicle distribution conditions, as well as on-the-way replenishment delivery modes. We constructed a model with the optimization objectives of minimizing delivery costs, carbon emissions, and maximizing the average freshness of perishable products delivered to customers. We designed a hybrid algorithm that combines large-scale neighborhood search with NSGA-II to solve the model. The experimental results are as follows: (1) An analysis of the number of depots revealed that increasing the number of depots can optimize the delivery costs, carbon emissions, and freshness to some extent. (2) An analysis of the on-the-way replenishment strategy indicated that the mode of on-the-way replenishment across depots outperforms the traditional delivery mode (without considering on-the-way replenishment) and the strategy that limits replenishment within depots. (3) Random simulation of road congestion index showed that the optimization objectives fluctuated within a normal range, indicating that the model developed in this study is stable under random traffic conditions. (4) Sensitivity analysis of the time interval length (representing the rate of traffic condition changes) revealed that more severe changes in traffic conditions led to a certain degree of reduction in optimization results. (5) Analysis of the sensitivity factor for the freshness preservation effort level in cold chain logistics showed that a higher level of preservation effort results in higher freshness of perishable products delivered to customers. Therefore, based on the above research results, the multi-depot and multi-vehicle cold chain delivery route optimization model considering road traffic conditions and on-the-way replenishment strategy can provide valuable insights for relevant industries. It can assist companies in making decisions and choosing appropriate delivery solutions by optimizing costs, carbon emissions, and customer satisfaction as objectives within a Pareto optimal solution set.

## 7 Outlook

Today, research related to urban traffic congestion has become a popular research direction among many scholars. In large and medium-sized cities, road traffic congestion is more common, but scholars’ research on the driving speeds under congested conditions remains superficial, with no in-depth measurement of vehicle speeds. In practice, factors such as road gradients, traffic accidents, weather conditions, and drivers all affect driving speeds. If future scholars could delve deeper into these factors to better define vehicle speeds under traffic congestion, it would make subsequent research in cold chain logistics distribution easier.

On the other hand, we have noticed that although our algorithm yields good results, but it is time-consuming. Integrating clustering algorithm concepts into multi-objective evolutionary algorithms may further reduce the running time of the algorithm.

## Supporting information

S1 Table (XLSX)
